# Crystal structure of 2-[9-(2-hy­droxy­phen­yl)-1,8-dioxo-1,2,3,4,5,6,7,8,9,10-deca­hydro­acridin-10-yl]acetic acid

**DOI:** 10.1107/S2056989015021611

**Published:** 2015-11-21

**Authors:** Mehmet Akkurt, Jerry P. Jasinski, Shaaban K. Mohamed, Omyma A. Abd Allah, Asmaa H. A. Tamam, Mustafa R. Albayati

**Affiliations:** a Department of Physics, Faculty of Sciences, Erciyes University, 38039 Kayseri, Turkey; b Department of Chemistry, Keene State College, 229 Main Street, Keene, NH 03435-2001, USA; c Chemistry and Environmental Division, Manchester Metropolitan University, Manchester M1 5GD, England; d Chemistry Department, Faculty of Science, Minia University, 61519 El-Minia, Egypt; e Chemistry Department, Faculty of Science, Sohag University, 82524 Sohag, Egypt; f Kirkuk University, College of Science, Department of Chemistry, Kirkuk, Iraq

**Keywords:** crystal structure, acridines, acetic acid, hydrogen bonding, C—H⋯π inter­actions

## Abstract

The title compound, C_21_H_21_NO_5_, crystallizes with two mol­ecules in the asymmetric unit. In each mol­ecule, the central 1,4-di­hydro­pyridine ring adopts a shallow sofa conformations (with the C atom bearing the phenol ring as the flap), whereas the pendant cyclo­hexene rings both have twisted-boat conformations. Each mol­ecule features an intra­molecular O—H⋯O hydrogen bond, which closes an *S*(8) ring. In the crystal, the mol­ecules are linked by O—H⋯O, C—H⋯O and C—H⋯π inter­actions, forming a three-dimensional network.

## Related literature   

For the industrial and pharmaceutical applications of acridine compounds, see: Szymanska *et al.* (2000 ▸); Fox & Chanon (1988 ▸); Groundwater & Munawar (1997 ▸); Cane *et al.* (1991 ▸).
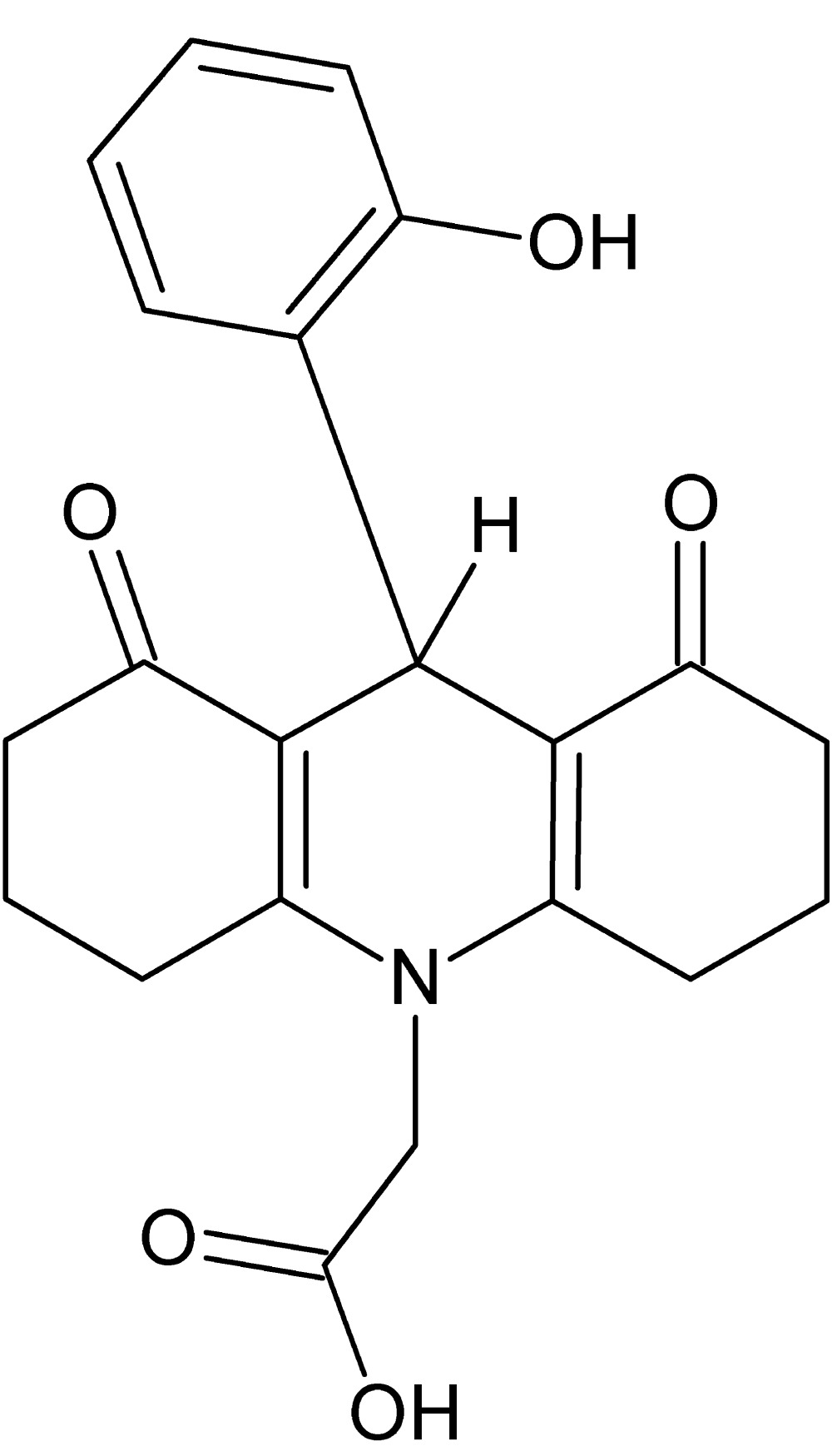


## Experimental   

### Crystal data  

C_21_H_21_NO_5_
*M* *_r_* = 367.39
Monoclinic, 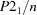

*a* = 19.4735 (7) Å
*b* = 8.9773 (4) Å
*c* = 20.3414 (8) Å
β = 91.619 (3)°
*V* = 3554.7 (2) Å^3^
*Z* = 8
Mo *K*α radiation
μ = 0.10 mm^−1^
*T* = 293 K
0.26 × 0.22 × 0.12 mm

### Data collection  

Agilent Xcalibur (Eos, Gemini) diffractometer
Absorption correction: multi-scan (*CrysAlis PRO*; Agilent, 2014 ▸) *T*_min_ = 0.901, *T*_max_ = 1.000
33119 measured reflections
11946 independent reflections
6442 reflections with *I* > 2σ(*I*)
*R*_int_ = 0.044

### Refinement  

*R*[*F* ^2^ > 2σ(*F* ^2^)] = 0.079
*wR*(*F* ^2^) = 0.229
*S* = 1.01
11946 reflections
499 parameters
7 restraints
?
Δρ_max_ = 0.36 e Å^−3^
Δρ_min_ = −0.36 e Å^−3^

### 

Data collection: *CrysAlis PRO* (Agilent, 2014 ▸); cell refinement: *CrysAlis PRO*; data reduction: *CrysAlis PRO*; program(s) used to solve structure: *SHELXS2014* (Sheldrick, 2008 ▸); program(s) used to refine structure: *SHELXL2014* (Sheldrick, 2015 ▸); molecular graphics: *ORTEP-3 for Windows* (Farrugia, 2012 ▸); software used to prepare material for publication: *PLATON* (Spek, 2009 ▸).

## Supplementary Material

Crystal structure: contains datablock(s) global, I. DOI: 10.1107/S2056989015021611/hb7542sup1.cif

Structure factors: contains datablock(s) I. DOI: 10.1107/S2056989015021611/hb7542Isup2.hkl

Supporting information file. DOI: 10.1107/S2056989015021611/hb7542Isup3.cml

**Fig. 1.**
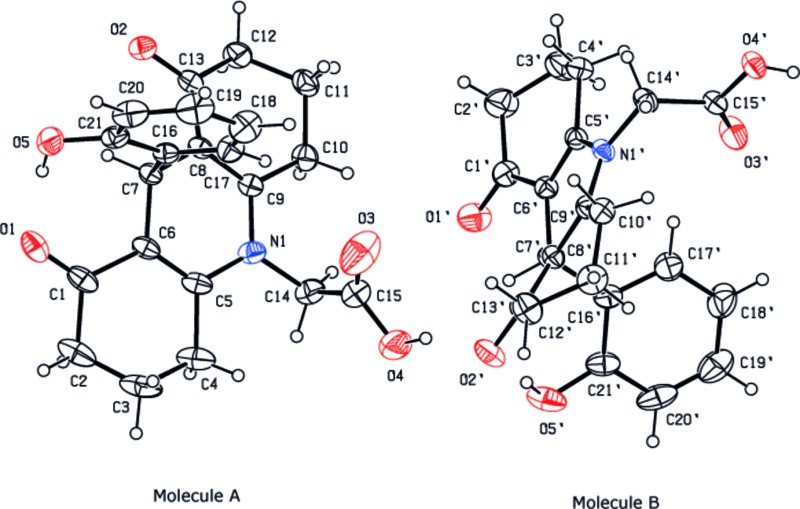
View of two molecules in the asymmetric unit of the title compound with displacement ellipsoids drawn at the 30% probability level.

. DOI: 10.1107/S2056989015021611/hb7542fig1.tif
View of two mol­ecules in the asymmetric unit of the title compound with displacement ellipsoids drawn at the 30% probability level.

**Fig. 2.**
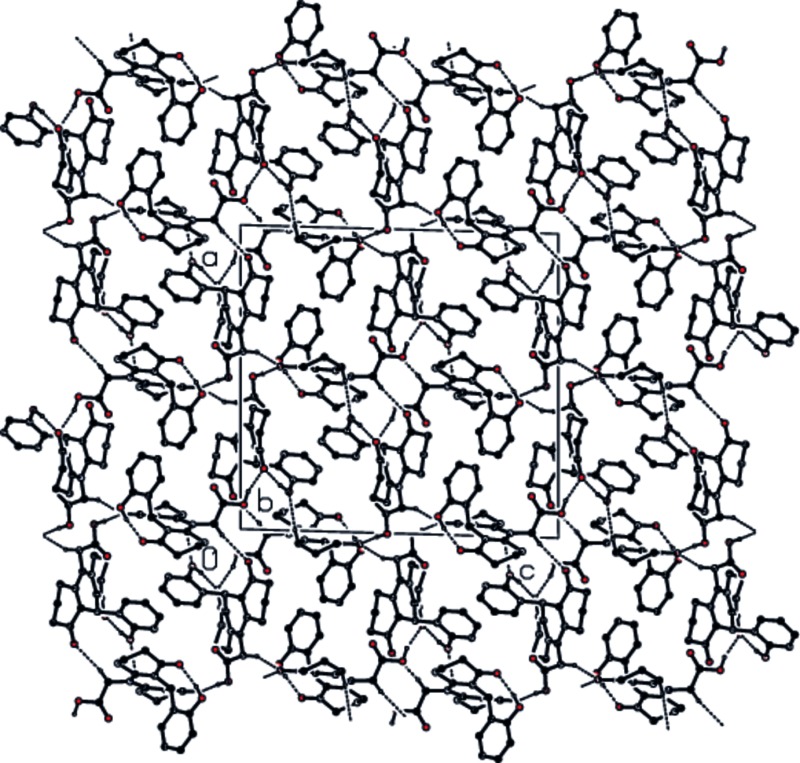
View of the hydrogen bonding and packing of the title compounds down the [010] axis. H atoms not involved in hydrogen bonding have been omitted for clarity.

. DOI: 10.1107/S2056989015021611/hb7542fig2.tif
View of the hydrogen bonding and packing of the title compounds down the [010] axis. H atoms not involved in hydrogen bonding have been omitted for clarity.

CCDC reference: 1437049

Additional supporting information:  crystallographic information; 3D view; checkCIF report

## Figures and Tables

**Table 1:** Hydrogen-bond geometry (Å, °)

*D*—H⋯*A*	*D*—H	H⋯*A*	*D*⋯*A*	*D*—H⋯*A*
O4—H4⋯O2′	0.88 (2)	1.84 (3)	2.683 (3)	162 (3)
O4′—H4′⋯O2^i^	0.84 (3)	1.85 (3)	2.673 (2)	165 (3)
O5—H5⋯O1	0.83 (3)	1.80 (3)	2.616 (3)	168 (3)
O5′—H5′⋯O2′	0.85 (4)	1.96 (4)	2.797 (3)	171 (4)
C10—H10*A*⋯O5′^ii^	0.97	2.60	3.380 (3)	138
C14—H14*B*⋯O1′^ii^	0.97	2.43	3.347 (3)	158
C14′—H14*D*⋯O2^iii^	0.97	2.36	3.241 (3)	151
C17′—H17′⋯O3′	0.93	2.49	3.326 (3)	149

Symmetry codes: (i) 
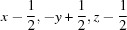
; (ii) 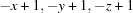; (iii) 
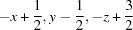
.

## supplementary crystallographic information

### S1. Comment

Acridines and acridinium salts are highly fluorescent (Szymanska *et al.*, 2000), and as electron acceptors in photochemical processes (Fox & Chanon, 1988). In addition, acridine derivatives have found application as antimalarial, and antitumour agents (Groundwater & Munawar, 1997; Cane *et al.*, 1991). In this context, we report herein the synthesis and crystal structure of the title compound.

Fig. 1 shows two molecules (A and B) of the title compound in the asymmetric unit. In the molecules (A and B), the central 1,4-dihydropyridine rings (N1/C5–C9 and N1'/C5'–C9') of the 1,2,3,4,5,6,7,8,9,10-decahydroacridine ring systems (N1/C1–C13 and N1'/C1'–C13') adopt nearly a chair conformation [the puckering parameters are Q_T_ = 0.261 (2) Å, θ = 110.0 (4) °, φ = 360.0 (5) ° and Q_T_ = 0.337 (2) Å, θ = 108.1 (3) °, φ = 4.8 (4) °, repsectively], which the cyclohexene rings (C1–C6, C8–C13 and C1'–C6', C8'–C13') of the 1,2,3,4,5,6,7,8,9,10-decahydroacridine ring systems have a twisted-boat conformation [for molecule A, the puckering parameters are Q_T_ = 0.470 (4) Å, θ = 120.0 (4) °, φ = 286.9 (4) ° and Q_T_ = 0.460 (3) Å, θ = 60.2 (4) °, φ = 196.0 (4) °, repsectively, and for molecule B, Q_T_ = 0.465 (3) Å, θ = 120.0 (4) °, φ = 288.3 (4) ° and Q_T_ = 0.474 (3) Å, θ = 61.3 (4) °, φ = 194.8 (4) °, respectively].

In the crystal structure, adjacent molecules are connected by O—H···O, C—H···O and C—H···π interactions, forming a three-dimensional network (Table 1, Fig. 2).

### S2. Experimental

A mixture of ethyl 2-[9-(2-hydroxyphenyl)-1,8-dioxo-2,3,4,5,6,7,8,9-octahydroacridin- 10(1*H*)-yl) acetate (2.0 g, 0.005 mol) and a solution of NaOH (0.4 g, 0.01 mol) in (40 ml) ethanol was heated under reflux for 5 h. The reaction mixture was poured onto cold water and acidified with conc. HCl. The separated solid was filtered off, dried and crystallized from ethanol to afford dark red prisms. Yield: 93.5%, mp. 511–513 K. IR (λ_max_, cm^-1^): 3356 (OH_acid_), 3119 (OH_arom._), 3081 (CH_arom._) 2969–2852 (CH_aliph._), 1726 (C=O_acid_), 1625 (C=O_cyclic ketone_). ^1^*H*-NMR (DMSO-d_6_), δ p.p.m.: 13.39 (s, 1H, OH acid, disappeared by D_2_O), 9.6 (s, 1H, OH_arom._, disappeared by D_2_O), 6.96–6.67 (m, 4H, CH_arom._), 4.99 (s, 1H, CH), 4.72 (s, 2H, CH_2_COOH), 2.91 (t, 2H, CH_2_C=O), 2.45 (t, 2H, CH_2_CO), 2.29 (t, 4H, 2CH_2_-C=C), 1.96 (m, 2H, CH_2_—CH_2_—CH_2_), 1.81 (m, 2H, CH_2_–CH_2_–CH_2_). ^13^C-NMR (DMSO-d_6_), δ p.p.m.: 197.62, 171.23, 156.26, 153.83, 132.81, 128.46, 127.77, 120.23, 117.26, 114.83, 47.93, 36.06, 25.99, 25.67.

### S3. Refinement

The hydroxyl hydrogen atoms were found from a difference Fourier map and the O—H distances were restrained to 0.82 (2) Å, using the *DFIX* option and included in the structure-factor calculations with *U*_iso_(H) = 1.5*U*_eq_(O). The remaining H atoms were placed in calculated positions with C—H = 0.93 - 0.98 Å, and refined as riding with *U*_iso_(H) = 1.2*U*_eq_(C).

### Crystal data

**Table:**

C_21_H_21_NO_5_	*F*(000) = 1552
*M**_r_* = 367.39	*D*_x_ = 1.373 Mg m^−^^3^
Monoclinic, *P*2_1_/*n*	Mo *K*α radiation, λ = 0.71073 Å
Hall symbol: -P 2yn	Cell parameters from 4652 reflections
*a* = 19.4735 (7) Å	θ = 3.8–27.1°
*b* = 8.9773 (4) Å	µ = 0.10 mm^−^^1^
*c* = 20.3414 (8) Å	*T* = 293 K
β = 91.619 (3)°	Prism, dark red
*V* = 3554.7 (2) Å^3^	0.26 × 0.22 × 0.12 mm
*Z* = 8	

### Data collection

**Table:**

Agilent Xcalibur (Eos, Gemini) diffractometer	11946 independent reflections
Radiation source: Enhance (Mo) X-ray Source	6442 reflections with *I* > 2σ(*I*)
Graphite monochromator	*R*_int_ = 0.044
Detector resolution: 16.0416 pixels mm^-1^	θ_max_ = 32.9°, θ_min_ = 2.9°
ω scans	*h* = −19→29
Absorption correction: multi-scan (*CrysAlis PRO*; Agilent, 2014)	*k* = −13→13
*T*_min_ = 0.901, *T*_max_ = 1.000	*l* = −30→30
33119 measured reflections	

### Refinement

**Table:**

Refinement on *F*^2^	7 restraints
Least-squares matrix: full	Hydrogen site location: mixed
*R*[*F*^2^ > 2σ(*F*^2^)] = 0.079	*w* = 1/[σ^2^(*F*_o_^2^) + (0.092*P*)^2^ + 1.4407*P*] where *P* = (*F*_o_^2^ + 2*F*_c_^2^)/3
*wR*(*F*^2^) = 0.229	(Δ/σ)_max_ < 0.001
*S* = 1.01	Δρ_max_ = 0.36 e Å^−^^3^
11946 reflections	Δρ_min_ = −0.36 e Å^−^^3^
499 parameters	

### Special details

**Table:**

Geometry. Bond distances, angles *etc*. have been calculated using the rounded fractional coordinates. All su's are estimated from the variances of the (full) variance-covariance matrix. The cell e.s.d.'s are taken into account in the estimation of distances, angles and torsion angles
Refinement. Refinement on *F*^2^ for ALL reflections except those flagged by the user for potential systematic errors. Weighted *R*-factors *wR* and all goodnesses of fit *S* are based on *F*^2^, conventional *R*-factors *R* are based on *F*, with *F* set to zero for negative *F*^2^. The observed criterion of *F*^2^ > σ(*F*^2^) is used only for calculating -*R*-factor-obs *etc*. and is not relevant to the choice of reflections for refinement. *R*-factors based on *F*^2^ are statistically about twice as large as those based on *F*, and *R*-factors based on ALL data will be even larger.

### Fractional atomic coordinates and isotropic or equivalent isotropic displacement parameters (Å^2^)

**Table:**

	*x*	*y*	*z*	*U*_iso_*/*U*_eq_	
O1	0.54951 (10)	1.0494 (2)	0.80712 (11)	0.0622 (7)	
O2	0.46153 (8)	0.56884 (17)	0.87478 (8)	0.0433 (5)	
O3	0.36550 (14)	0.6857 (4)	0.58385 (13)	0.1172 (15)	
O4	0.41939 (12)	0.6805 (4)	0.49001 (11)	0.0891 (9)	
O5	0.44030 (11)	1.0230 (2)	0.87818 (9)	0.0565 (6)	
N1	0.48876 (10)	0.6798 (2)	0.65174 (9)	0.0445 (6)	
C1	0.54926 (12)	1.0057 (3)	0.75006 (15)	0.0496 (8)	
O1'	0.39287 (9)	0.2869 (3)	0.52414 (10)	0.0675 (8)	
C2	0.59167 (17)	1.0827 (4)	0.69950 (18)	0.0732 (13)	
O2'	0.29880 (9)	0.7291 (2)	0.42658 (10)	0.0601 (7)	
C3	0.5597 (2)	1.0692 (4)	0.63282 (18)	0.0815 (15)	
O3'	0.10134 (9)	0.1149 (2)	0.42055 (10)	0.0653 (7)	
C4	0.54754 (17)	0.9082 (3)	0.61352 (15)	0.0661 (10)	
O4'	−0.00242 (8)	0.1429 (2)	0.46142 (8)	0.0472 (5)	
C5	0.51323 (12)	0.8215 (3)	0.66776 (12)	0.0446 (7)	
O5'	0.37299 (12)	0.5356 (3)	0.34753 (12)	0.0773 (9)	
C6	0.50975 (11)	0.8746 (2)	0.72947 (11)	0.0401 (7)	
C7	0.46943 (10)	0.7948 (2)	0.78090 (10)	0.0336 (6)	
C8	0.46571 (10)	0.6316 (2)	0.76357 (10)	0.0341 (6)	
C9	0.47006 (11)	0.5828 (2)	0.70101 (10)	0.0366 (6)	
C10	0.45820 (13)	0.4225 (3)	0.68218 (12)	0.0473 (8)	
C11	0.42648 (14)	0.3332 (3)	0.73625 (13)	0.0524 (8)	
C12	0.46099 (16)	0.3626 (3)	0.80162 (13)	0.0533 (8)	
C13	0.46154 (10)	0.5261 (2)	0.81698 (11)	0.0360 (6)	
C14	0.48528 (14)	0.6324 (3)	0.58306 (12)	0.0538 (9)	
C15	0.41689 (17)	0.6716 (4)	0.55438 (14)	0.0623 (10)	
C16	0.39868 (11)	0.8636 (2)	0.78994 (10)	0.0355 (6)	
C17	0.34257 (12)	0.8207 (3)	0.75127 (12)	0.0479 (8)	
C18	0.27772 (14)	0.8762 (4)	0.76125 (15)	0.0654 (10)	
C19	0.26799 (16)	0.9772 (4)	0.81090 (16)	0.0682 (11)	
C20	0.32242 (17)	1.0251 (3)	0.84917 (14)	0.0609 (10)	
C21	0.38832 (13)	0.9711 (3)	0.83864 (11)	0.0438 (7)	
N1'	0.15357 (8)	0.3463 (2)	0.49752 (8)	0.0362 (5)	
C1'	0.33538 (12)	0.2426 (3)	0.53659 (12)	0.0468 (8)	
C2'	0.32463 (16)	0.1296 (4)	0.58950 (16)	0.0689 (11)	
C3'	0.26161 (16)	0.0409 (4)	0.57678 (18)	0.0716 (11)	
C4'	0.19809 (13)	0.1389 (3)	0.56772 (12)	0.0491 (8)	
C5'	0.21077 (11)	0.2651 (2)	0.52069 (10)	0.0360 (6)	
C6'	0.27448 (10)	0.3044 (2)	0.50276 (10)	0.0361 (6)	
C7'	0.28481 (10)	0.4197 (2)	0.45054 (10)	0.0365 (6)	
C8'	0.22693 (10)	0.5304 (2)	0.45338 (10)	0.0352 (6)	
C9'	0.16270 (10)	0.4853 (2)	0.46978 (10)	0.0336 (6)	
C10'	0.10140 (11)	0.5844 (3)	0.46075 (11)	0.0442 (7)	
C11'	0.11568 (13)	0.7190 (3)	0.41893 (14)	0.0550 (9)	
C12'	0.18185 (14)	0.7926 (3)	0.43997 (15)	0.0580 (9)	
C13'	0.24005 (12)	0.6846 (3)	0.43923 (11)	0.0428 (7)	
C14'	0.08462 (11)	0.2878 (3)	0.50771 (11)	0.0412 (7)	
C15'	0.06362 (11)	0.1735 (2)	0.45782 (11)	0.0382 (6)	
C16'	0.28966 (11)	0.3533 (3)	0.38153 (11)	0.0422 (7)	
C17'	0.24941 (14)	0.2339 (4)	0.36209 (13)	0.0594 (9)	
C18'	0.25191 (18)	0.1744 (5)	0.29965 (15)	0.0773 (13)	
C19'	0.29600 (19)	0.2327 (5)	0.25536 (15)	0.0817 (13)	
C20'	0.33619 (17)	0.3501 (4)	0.27253 (14)	0.0710 (13)	
C21'	0.33296 (13)	0.4147 (3)	0.33476 (13)	0.0538 (9)	
H2A	0.59640	1.18720	0.71090	0.0880*	
H2B	0.63720	1.03910	0.69970	0.0880*	
H3A	0.58940	1.11560	0.60120	0.0980*	
H3B	0.51620	1.12200	0.63150	0.0980*	
H4	0.3763 (10)	0.686 (4)	0.4766 (17)	0.0850*	
H4A	0.59110	0.86180	0.60400	0.0790*	
H4B	0.51870	0.90450	0.57390	0.0790*	
H5	0.4779 (12)	1.025 (4)	0.8603 (16)	0.0850*	
H7	0.49510	0.80370	0.82280	0.0400*	
H10A	0.50170	0.37770	0.67120	0.0570*	
H10B	0.42820	0.41860	0.64330	0.0570*	
H11A	0.37810	0.35810	0.73850	0.0630*	
H11B	0.42990	0.22800	0.72610	0.0630*	
H12A	0.50780	0.32580	0.80130	0.0640*	
H12B	0.43700	0.30960	0.83560	0.0640*	
H14A	0.49240	0.52570	0.58030	0.0640*	
H14B	0.52100	0.68150	0.55880	0.0640*	
H17	0.34880	0.75250	0.71760	0.0570*	
H18	0.24080	0.84560	0.73460	0.0780*	
H19	0.22410	1.01310	0.81850	0.0820*	
H20	0.31550	1.09410	0.88240	0.0730*	
H2'1	0.36400	0.06330	0.59220	0.0830*	
H2'2	0.32130	0.18000	0.63150	0.0830*	
H3'1	0.25500	−0.02650	0.61330	0.0860*	
H4'	−0.013 (2)	0.088 (4)	0.4291 (15)	0.1160*	
H3'2	0.26720	−0.01870	0.53750	0.0860*	
H5'	0.353 (2)	0.590 (4)	0.3751 (18)	0.1160*	
H4'1	0.16000	0.07900	0.55100	0.0590*	
H4'2	0.18560	0.17920	0.61000	0.0590*	
H7'	0.32790	0.47230	0.46090	0.0440*	
H10C	0.08660	0.61730	0.50350	0.0530*	
H10D	0.06410	0.52780	0.44040	0.0530*	
H11C	0.11810	0.68890	0.37330	0.0660*	
H11D	0.07830	0.78970	0.42240	0.0660*	
H12C	0.19110	0.87480	0.41060	0.0700*	
H12D	0.17770	0.83260	0.48400	0.0700*	
H14C	0.05200	0.36940	0.50590	0.0490*	
H14D	0.08310	0.24390	0.55120	0.0490*	
H17'	0.21970	0.19230	0.39200	0.0710*	
H18'	0.22370	0.09470	0.28780	0.0930*	
H19'	0.29840	0.19180	0.21350	0.0980*	
H20'	0.36650	0.38840	0.24230	0.0850*	

### Atomic displacement parameters (Å^2^)

**Table:**

	*U*^11^	*U*^22^	*U*^33^	*U*^12^	*U*^13^	*U*^23^
O1	0.0625 (12)	0.0388 (10)	0.0849 (14)	−0.0124 (9)	−0.0024 (10)	−0.0031 (10)
O2	0.0532 (9)	0.0330 (8)	0.0441 (8)	0.0069 (7)	0.0067 (7)	0.0040 (7)
O3	0.0716 (16)	0.201 (4)	0.0788 (16)	0.0340 (19)	−0.0036 (13)	−0.010 (2)
O4	0.0745 (15)	0.130 (2)	0.0619 (13)	−0.0260 (16)	−0.0121 (11)	0.0250 (14)
O5	0.0814 (13)	0.0385 (9)	0.0495 (10)	−0.0029 (10)	0.0027 (9)	−0.0070 (8)
N1	0.0530 (11)	0.0409 (11)	0.0402 (10)	0.0023 (9)	0.0120 (8)	0.0023 (8)
C1	0.0378 (12)	0.0308 (12)	0.0806 (18)	0.0008 (10)	0.0074 (11)	0.0099 (12)
O1'	0.0364 (9)	0.0949 (17)	0.0712 (12)	0.0032 (10)	0.0009 (8)	0.0210 (11)
C2	0.0665 (19)	0.0437 (16)	0.111 (3)	−0.0115 (14)	0.0289 (18)	0.0119 (16)
O2'	0.0464 (10)	0.0474 (11)	0.0862 (13)	−0.0130 (8)	−0.0034 (9)	0.0174 (10)
C3	0.102 (3)	0.0479 (17)	0.097 (3)	−0.0075 (17)	0.048 (2)	0.0218 (17)
O3'	0.0486 (10)	0.0677 (13)	0.0806 (13)	−0.0091 (9)	0.0210 (9)	−0.0326 (11)
C4	0.0743 (19)	0.0516 (17)	0.0743 (18)	0.0038 (15)	0.0354 (15)	0.0169 (14)
O4'	0.0376 (8)	0.0464 (10)	0.0579 (10)	−0.0082 (7)	0.0069 (7)	−0.0111 (8)
C5	0.0420 (12)	0.0351 (12)	0.0575 (14)	0.0033 (10)	0.0141 (10)	0.0122 (10)
O5'	0.0692 (13)	0.0725 (16)	0.0921 (16)	−0.0095 (12)	0.0374 (11)	0.0162 (12)
C6	0.0364 (11)	0.0285 (10)	0.0558 (13)	0.0020 (9)	0.0093 (9)	0.0064 (9)
C7	0.0352 (10)	0.0258 (10)	0.0398 (10)	−0.0001 (8)	0.0031 (8)	0.0020 (8)
C8	0.0320 (10)	0.0264 (10)	0.0441 (11)	0.0012 (8)	0.0062 (8)	0.0020 (8)
C9	0.0349 (10)	0.0320 (11)	0.0430 (11)	0.0026 (9)	0.0055 (8)	0.0022 (9)
C10	0.0545 (14)	0.0370 (12)	0.0507 (13)	0.0011 (11)	0.0063 (10)	−0.0064 (10)
C11	0.0601 (15)	0.0346 (13)	0.0624 (15)	−0.0088 (11)	0.0025 (12)	−0.0014 (11)
C12	0.0791 (18)	0.0262 (11)	0.0546 (14)	−0.0015 (12)	0.0040 (12)	0.0034 (10)
C13	0.0316 (10)	0.0284 (10)	0.0482 (12)	0.0025 (8)	0.0054 (8)	0.0035 (9)
C14	0.0625 (16)	0.0557 (16)	0.0435 (13)	0.0037 (13)	0.0095 (11)	0.0027 (11)
C15	0.0685 (19)	0.070 (2)	0.0484 (15)	−0.0049 (16)	0.0012 (13)	0.0032 (13)
C16	0.0408 (11)	0.0287 (10)	0.0375 (10)	0.0056 (9)	0.0088 (8)	0.0046 (8)
C17	0.0428 (12)	0.0545 (15)	0.0466 (12)	0.0086 (11)	0.0054 (10)	−0.0047 (11)
C18	0.0434 (14)	0.086 (2)	0.0669 (17)	0.0169 (15)	0.0031 (12)	0.0012 (16)
C19	0.0555 (17)	0.072 (2)	0.078 (2)	0.0292 (16)	0.0182 (15)	0.0098 (16)
C20	0.082 (2)	0.0421 (14)	0.0599 (16)	0.0219 (14)	0.0268 (14)	0.0026 (12)
C21	0.0616 (14)	0.0294 (11)	0.0409 (11)	0.0049 (10)	0.0095 (10)	0.0052 (9)
N1'	0.0292 (8)	0.0374 (10)	0.0422 (9)	−0.0049 (7)	0.0069 (7)	−0.0046 (7)
C1'	0.0410 (12)	0.0535 (15)	0.0459 (12)	0.0038 (11)	0.0018 (9)	0.0020 (11)
C2'	0.0591 (17)	0.075 (2)	0.0720 (19)	0.0043 (16)	−0.0084 (14)	0.0261 (16)
C3'	0.0713 (19)	0.0569 (18)	0.086 (2)	−0.0039 (15)	−0.0069 (16)	0.0285 (16)
C4'	0.0536 (14)	0.0469 (14)	0.0470 (13)	−0.0098 (12)	0.0071 (10)	0.0056 (11)
C5'	0.0376 (11)	0.0336 (11)	0.0369 (10)	−0.0030 (9)	0.0050 (8)	−0.0028 (8)
C6'	0.0345 (10)	0.0365 (11)	0.0375 (10)	0.0010 (9)	0.0046 (8)	−0.0014 (8)
C7'	0.0286 (9)	0.0361 (11)	0.0449 (11)	−0.0009 (9)	0.0040 (8)	0.0021 (9)
C8'	0.0315 (10)	0.0346 (11)	0.0395 (10)	−0.0018 (8)	0.0023 (8)	−0.0017 (8)
C9'	0.0326 (10)	0.0350 (11)	0.0334 (10)	−0.0015 (8)	0.0025 (7)	−0.0066 (8)
C10'	0.0339 (11)	0.0530 (14)	0.0458 (12)	0.0058 (10)	0.0029 (9)	−0.0017 (10)
C11'	0.0497 (14)	0.0535 (16)	0.0618 (15)	0.0157 (12)	0.0008 (11)	0.0033 (12)
C12'	0.0601 (16)	0.0362 (13)	0.0775 (18)	0.0065 (12)	−0.0011 (13)	−0.0008 (12)
C13'	0.0394 (12)	0.0369 (12)	0.0519 (13)	−0.0039 (10)	−0.0029 (9)	0.0011 (10)
C14'	0.0305 (10)	0.0420 (12)	0.0517 (12)	−0.0055 (9)	0.0109 (9)	−0.0070 (10)
C15'	0.0343 (10)	0.0329 (11)	0.0478 (12)	−0.0022 (9)	0.0065 (9)	−0.0018 (9)
C16'	0.0328 (10)	0.0492 (14)	0.0452 (12)	0.0106 (10)	0.0112 (9)	0.0049 (10)
C17'	0.0534 (15)	0.0712 (19)	0.0543 (15)	−0.0054 (14)	0.0167 (12)	−0.0141 (13)
C18'	0.074 (2)	0.098 (3)	0.0604 (17)	−0.0054 (19)	0.0126 (15)	−0.0292 (17)
C19'	0.087 (2)	0.108 (3)	0.0509 (17)	0.018 (2)	0.0158 (16)	−0.0081 (18)
C20'	0.0696 (19)	0.093 (3)	0.0521 (16)	0.0293 (19)	0.0298 (14)	0.0192 (16)
C21'	0.0456 (13)	0.0590 (17)	0.0575 (15)	0.0118 (12)	0.0157 (11)	0.0162 (12)

### Geometric parameters (Å, º)

**Table:**

O1—C1	1.225 (4)	C11—H11A	0.9700
O2—C13	1.237 (3)	C11—H11B	0.9700
O3—C15	1.188 (4)	C12—H12A	0.9700
O4—C15	1.314 (4)	C12—H12B	0.9700
O5—C21	1.357 (3)	C14—H14A	0.9700
N1—C5	1.394 (3)	C14—H14B	0.9700
N1—C9	1.384 (3)	C17—H17	0.9300
N1—C14	1.460 (3)	C18—H18	0.9300
O4—H4	0.88 (2)	C19—H19	0.9300
O5—H5	0.83 (3)	C20—H20	0.9300
C1—C6	1.461 (3)	C1'—C6'	1.463 (3)
C1—C2	1.505 (4)	C1'—C2'	1.498 (4)
O1'—C1'	1.221 (3)	C2'—C3'	1.480 (5)
C2—C3	1.481 (5)	C3'—C4'	1.525 (4)
O2'—C13'	1.246 (3)	C4'—C5'	1.508 (3)
C3—C4	1.515 (5)	C5'—C6'	1.350 (3)
O3'—C15'	1.193 (3)	C6'—C7'	1.501 (3)
C4—C5	1.520 (4)	C7'—C16'	1.531 (3)
O4'—C15'	1.319 (3)	C7'—C8'	1.505 (3)
C5—C6	1.346 (3)	C8'—C13'	1.438 (3)
O5'—C21'	1.357 (4)	C8'—C9'	1.365 (3)
C6—C7	1.507 (3)	C9'—C10'	1.496 (3)
C7—C16	1.526 (3)	C10'—C11'	1.508 (4)
C7—C8	1.508 (3)	C11'—C12'	1.500 (4)
C8—C9	1.351 (3)	C12'—C13'	1.492 (4)
C8—C13	1.445 (3)	C14'—C15'	1.492 (3)
C9—C10	1.505 (3)	C16'—C21'	1.402 (3)
C10—C11	1.507 (4)	C16'—C17'	1.379 (4)
C11—C12	1.496 (4)	C17'—C18'	1.380 (4)
C12—C13	1.501 (3)	C18'—C19'	1.366 (5)
C14—C15	1.481 (4)	C19'—C20'	1.353 (5)
C16—C17	1.383 (3)	C20'—C21'	1.395 (4)
C16—C21	1.402 (3)	C2'—H2'1	0.9700
C17—C18	1.378 (4)	C2'—H2'2	0.9700
C18—C19	1.374 (5)	C3'—H3'1	0.9700
C19—C20	1.367 (4)	C3'—H3'2	0.9700
C20—C21	1.394 (4)	C4'—H4'1	0.9700
N1'—C5'	1.402 (3)	C4'—H4'2	0.9700
N1'—C9'	1.383 (3)	C7'—H7'	0.9800
N1'—C14'	1.462 (3)	C10'—H10C	0.9700
C2—H2A	0.9700	C10'—H10D	0.9700
C2—H2B	0.9700	C11'—H11C	0.9700
C3—H3A	0.9700	C11'—H11D	0.9700
C3—H3B	0.9700	C12'—H12C	0.9700
C4—H4A	0.9700	C12'—H12D	0.9700
C4—H4B	0.9700	C14'—H14C	0.9700
O4'—H4'	0.84 (3)	C14'—H14D	0.9700
O5'—H5'	0.85 (4)	C17'—H17'	0.9300
C7—H7	0.9800	C18'—H18'	0.9300
C10—H10B	0.9700	C19'—H19'	0.9300
C10—H10A	0.9700	C20'—H20'	0.9300
			
C5—N1—C9	120.01 (18)	C17—C18—H18	120.00
C5—N1—C14	119.74 (19)	C20—C19—H19	120.00
C9—N1—C14	120.22 (18)	C18—C19—H19	120.00
C15—O4—H4	105 (2)	C19—C20—H20	120.00
C21—O5—H5	114 (2)	C21—C20—H20	120.00
C2—C1—C6	118.0 (3)	C2'—C1'—C6'	117.8 (2)
O1—C1—C2	120.9 (3)	O1'—C1'—C2'	121.1 (2)
O1—C1—C6	121.2 (2)	O1'—C1'—C6'	121.0 (2)
C1—C2—C3	111.3 (3)	C1'—C2'—C3'	111.8 (3)
C2—C3—C4	112.0 (3)	C2'—C3'—C4'	112.1 (3)
C3—C4—C5	111.7 (3)	C3'—C4'—C5'	111.3 (2)
N1—C5—C4	116.9 (2)	N1'—C5'—C4'	117.47 (19)
N1—C5—C6	121.0 (2)	N1'—C5'—C6'	120.06 (17)
C4—C5—C6	122.0 (2)	C4'—C5'—C6'	122.42 (19)
C1—C6—C5	120.8 (2)	C1'—C6'—C5'	120.84 (19)
C5—C6—C7	121.33 (19)	C5'—C6'—C7'	120.94 (18)
C1—C6—C7	117.7 (2)	C1'—C6'—C7'	118.12 (18)
C8—C7—C16	112.57 (16)	C8'—C7'—C16'	110.98 (17)
C6—C7—C16	112.41 (16)	C6'—C7'—C16'	113.18 (17)
C6—C7—C8	108.79 (16)	C6'—C7'—C8'	108.23 (16)
C7—C8—C9	122.10 (17)	C7'—C8'—C9'	120.39 (16)
C7—C8—C13	117.66 (17)	C7'—C8'—C13'	119.37 (18)
C9—C8—C13	120.13 (17)	C9'—C8'—C13'	120.24 (18)
N1—C9—C8	120.19 (17)	N1'—C9'—C8'	119.77 (17)
C8—C9—C10	122.45 (18)	C8'—C9'—C10'	121.81 (18)
N1—C9—C10	117.31 (18)	N1'—C9'—C10'	118.38 (18)
C9—C10—C11	112.7 (2)	C9'—C10'—C11'	112.74 (18)
C10—C11—C12	111.8 (2)	C10'—C11'—C12'	111.3 (2)
C11—C12—C13	111.0 (2)	C11'—C12'—C13'	111.0 (2)
O2—C13—C12	120.08 (19)	O2'—C13'—C12'	119.7 (2)
O2—C13—C8	120.85 (17)	O2'—C13'—C8'	121.3 (2)
C8—C13—C12	118.99 (19)	C8'—C13'—C12'	119.0 (2)
N1—C14—C15	108.9 (2)	N1'—C14'—C15'	112.69 (18)
O3—C15—C14	126.0 (3)	O3'—C15'—C14'	125.0 (2)
O4—C15—C14	110.4 (3)	O4'—C15'—C14'	110.75 (18)
O3—C15—O4	123.5 (3)	O3'—C15'—O4'	124.2 (2)
C17—C16—C21	117.8 (2)	C17'—C16'—C21'	117.3 (2)
C7—C16—C17	121.36 (18)	C7'—C16'—C17'	121.1 (2)
C7—C16—C21	120.82 (19)	C7'—C16'—C21'	121.6 (2)
C16—C17—C18	121.9 (2)	C16'—C17'—C18'	122.0 (3)
C17—C18—C19	119.5 (3)	C17'—C18'—C19'	119.9 (4)
C18—C19—C20	120.4 (3)	C18'—C19'—C20'	119.9 (3)
C19—C20—C21	120.4 (3)	C19'—C20'—C21'	121.2 (3)
C16—C21—C20	120.0 (2)	C16'—C21'—C20'	119.7 (3)
O5—C21—C16	122.4 (2)	O5'—C21'—C16'	122.5 (2)
O5—C21—C20	117.6 (2)	O5'—C21'—C20'	117.8 (3)
C5'—N1'—C9'	119.76 (16)	C1'—C2'—H2'1	109.00
C5'—N1'—C14'	119.33 (18)	C1'—C2'—H2'2	109.00
C9'—N1'—C14'	120.74 (17)	C3'—C2'—H2'1	109.00
C3—C2—H2B	109.00	C3'—C2'—H2'2	109.00
H2A—C2—H2B	108.00	H2'1—C2'—H2'2	108.00
C1—C2—H2A	109.00	C2'—C3'—H3'1	109.00
C1—C2—H2B	109.00	C2'—C3'—H3'2	109.00
C3—C2—H2A	109.00	C4'—C3'—H3'1	109.00
C2—C3—H3A	109.00	C4'—C3'—H3'2	109.00
C2—C3—H3B	109.00	H3'1—C3'—H3'2	108.00
C4—C3—H3A	109.00	C3'—C4'—H4'1	109.00
C4—C3—H3B	109.00	C3'—C4'—H4'2	109.00
H3A—C3—H3B	108.00	C5'—C4'—H4'1	109.00
C3—C4—H4B	109.00	C5'—C4'—H4'2	109.00
H4A—C4—H4B	108.00	H4'1—C4'—H4'2	108.00
C5—C4—H4A	109.00	C6'—C7'—H7'	108.00
C5—C4—H4B	109.00	C8'—C7'—H7'	108.00
C3—C4—H4A	109.00	C16'—C7'—H7'	108.00
C15'—O4'—H4'	107 (3)	C9'—C10'—H10C	109.00
C21'—O5'—H5'	108 (3)	C9'—C10'—H10D	109.00
C8—C7—H7	108.00	C11'—C10'—H10C	109.00
C16—C7—H7	108.00	C11'—C10'—H10D	109.00
C6—C7—H7	108.00	H10C—C10'—H10D	108.00
C9—C10—H10B	109.00	C10'—C11'—H11C	109.00
C9—C10—H10A	109.00	C10'—C11'—H11D	109.00
H10A—C10—H10B	108.00	C12'—C11'—H11C	109.00
C11—C10—H10A	109.00	C12'—C11'—H11D	109.00
C11—C10—H10B	109.00	H11C—C11'—H11D	108.00
C10—C11—H11A	109.00	C11'—C12'—H12C	109.00
H11A—C11—H11B	108.00	C11'—C12'—H12D	109.00
C12—C11—H11B	109.00	C13'—C12'—H12C	110.00
C10—C11—H11B	109.00	C13'—C12'—H12D	109.00
C12—C11—H11A	109.00	H12C—C12'—H12D	108.00
C13—C12—H12A	109.00	N1'—C14'—H14C	109.00
C11—C12—H12B	109.00	N1'—C14'—H14D	109.00
C11—C12—H12A	109.00	C15'—C14'—H14C	109.00
C13—C12—H12B	109.00	C15'—C14'—H14D	109.00
H12A—C12—H12B	108.00	H14C—C14'—H14D	108.00
C15—C14—H14A	110.00	C16'—C17'—H17'	119.00
C15—C14—H14B	110.00	C18'—C17'—H17'	119.00
N1—C14—H14B	110.00	C17'—C18'—H18'	120.00
N1—C14—H14A	110.00	C19'—C18'—H18'	120.00
H14A—C14—H14B	108.00	C18'—C19'—H19'	120.00
C16—C17—H17	119.00	C20'—C19'—H19'	120.00
C18—C17—H17	119.00	C19'—C20'—H20'	119.00
C19—C18—H18	120.00	C21'—C20'—H20'	119.00
			
C5—N1—C9—C8	9.8 (3)	C5'—N1'—C9'—C8'	12.7 (3)
C14—N1—C9—C8	−172.4 (2)	C14'—N1'—C9'—C8'	−172.10 (19)
C5—N1—C14—C15	−90.8 (3)	C5'—N1'—C14'—C15'	−81.6 (2)
C9—N1—C14—C15	91.4 (3)	C9'—N1'—C14'—C15'	103.3 (2)
C14—N1—C5—C4	−11.2 (3)	C14'—N1'—C5'—C4'	−13.1 (3)
C9—N1—C5—C6	−9.8 (3)	C9'—N1'—C5'—C6'	−15.4 (3)
C14—N1—C5—C6	172.4 (2)	C14'—N1'—C5'—C6'	169.37 (19)
C9—N1—C5—C4	166.6 (2)	C9'—N1'—C5'—C4'	162.15 (19)
C5—N1—C9—C10	−167.6 (2)	C5'—N1'—C9'—C10'	−165.21 (18)
C14—N1—C9—C10	10.2 (3)	C14'—N1'—C9'—C10'	10.0 (3)
C6—C1—C2—C3	31.3 (4)	C6'—C1'—C2'—C3'	30.4 (4)
O1—C1—C2—C3	−150.7 (3)	O1'—C1'—C2'—C3'	−152.9 (3)
C2—C1—C6—C5	2.3 (3)	C2'—C1'—C6'—C5'	2.7 (3)
C2—C1—C6—C7	177.9 (2)	C2'—C1'—C6'—C7'	178.9 (2)
O1—C1—C6—C5	−175.7 (2)	O1'—C1'—C6'—C5'	−174.0 (2)
O1—C1—C6—C7	−0.1 (3)	O1'—C1'—C6'—C7'	2.2 (3)
C1—C2—C3—C4	−56.1 (4)	C1'—C2'—C3'—C4'	−55.5 (4)
C2—C3—C4—C5	47.8 (4)	C2'—C3'—C4'—C5'	47.8 (3)
C3—C4—C5—N1	169.3 (2)	C3'—C4'—C5'—N1'	167.6 (2)
C3—C4—C5—C6	−14.3 (4)	C3'—C4'—C5'—C6'	−14.9 (3)
N1—C5—C6—C1	165.4 (2)	N1'—C5'—C6'—C1'	167.06 (19)
C4—C5—C6—C1	−10.9 (4)	C4'—C5'—C6'—C1'	−10.4 (3)
N1—C5—C6—C7	−10.0 (3)	N1'—C5'—C6'—C7'	−9.1 (3)
C4—C5—C6—C7	173.7 (2)	C4'—C5'—C6'—C7'	173.51 (19)
C5—C6—C7—C16	−99.3 (2)	C5'—C6'—C7'—C16'	−91.8 (2)
C5—C6—C7—C8	26.1 (3)	C5'—C6'—C7'—C8'	31.6 (2)
C1—C6—C7—C8	−149.37 (19)	C1'—C6'—C7'—C8'	−144.58 (19)
C1—C6—C7—C16	85.3 (2)	C1'—C6'—C7'—C16'	92.0 (2)
C6—C7—C8—C9	−26.3 (3)	C6'—C7'—C8'—C9'	−34.2 (3)
C8—C7—C16—C17	−36.3 (3)	C8'—C7'—C16'—C17'	−84.2 (3)
C8—C7—C16—C21	142.7 (2)	C8'—C7'—C16'—C21'	93.2 (2)
C6—C7—C8—C13	149.89 (18)	C6'—C7'—C8'—C13'	145.26 (19)
C16—C7—C8—C9	99.0 (2)	C16'—C7'—C8'—C9'	90.6 (2)
C6—C7—C16—C21	−94.1 (2)	C6'—C7'—C16'—C21'	−144.9 (2)
C6—C7—C16—C17	87.0 (2)	C6'—C7'—C16'—C17'	37.7 (3)
C16—C7—C8—C13	−84.8 (2)	C16'—C7'—C8'—C13'	−90.0 (2)
C7—C8—C9—N1	10.0 (3)	C7'—C8'—C9'—N1'	14.1 (3)
C9—C8—C13—O2	175.8 (2)	C9'—C8'—C13'—O2'	177.3 (2)
C7—C8—C13—C12	−177.3 (2)	C7'—C8'—C13'—C12'	178.0 (2)
C13—C8—C9—N1	−166.08 (19)	C13'—C8'—C9'—N1'	−165.37 (19)
C9—C8—C13—C12	−1.0 (3)	C9'—C8'—C13'—C12'	−2.6 (3)
C7—C8—C9—C10	−172.69 (19)	C7'—C8'—C9'—C10'	−168.08 (19)
C7—C8—C13—O2	−0.5 (3)	C7'—C8'—C13'—O2'	−2.2 (3)
C13—C8—C9—C10	11.2 (3)	C13'—C8'—C9'—C10'	12.5 (3)
C8—C9—C10—C11	12.6 (3)	C8'—C9'—C10'—C11'	12.9 (3)
N1—C9—C10—C11	−170.1 (2)	N1'—C9'—C10'—C11'	−169.3 (2)
C9—C10—C11—C12	−45.5 (3)	C9'—C10'—C11'—C12'	−46.7 (3)
C10—C11—C12—C13	54.6 (3)	C10'—C11'—C12'—C13'	55.5 (3)
C11—C12—C13—C8	−32.0 (3)	C11'—C12'—C13'—C8'	−31.6 (3)
C11—C12—C13—O2	151.2 (2)	C11'—C12'—C13'—O2'	148.6 (2)
N1—C14—C15—O3	−26.1 (5)	N1'—C14'—C15'—O3'	12.6 (3)
N1—C14—C15—O4	157.7 (3)	N1'—C14'—C15'—O4'	−168.95 (18)
C7—C16—C17—C18	176.5 (2)	C7'—C16'—C17'—C18'	178.6 (3)
C21—C16—C17—C18	−2.5 (4)	C21'—C16'—C17'—C18'	1.1 (4)
C7—C16—C21—O5	2.4 (3)	C7'—C16'—C21'—O5'	0.1 (4)
C7—C16—C21—C20	−175.6 (2)	C7'—C16'—C21'—C20'	179.6 (2)
C17—C16—C21—O5	−178.6 (2)	C17'—C16'—C21'—O5'	177.6 (3)
C17—C16—C21—C20	3.5 (3)	C17'—C16'—C21'—C20'	−2.9 (4)
C16—C17—C18—C19	0.0 (5)	C16'—C17'—C18'—C19'	1.0 (5)
C17—C18—C19—C20	1.5 (5)	C17'—C18'—C19'—C20'	−1.1 (6)
C18—C19—C20—C21	−0.6 (5)	C18'—C19'—C20'—C21'	−0.8 (6)
C19—C20—C21—O5	179.9 (3)	C19'—C20'—C21'—O5'	−177.6 (3)
C19—C20—C21—C16	−2.0 (4)	C19'—C20'—C21'—C16'	2.9 (5)

### Hydrogen-bond geometry (Å, º)

**Table:**

*D*—H···*A*	*D*—H	H···*A*	*D*···*A*	*D*—H···*A*
O4—H4···O2′	0.88 (2)	1.84 (3)	2.683 (3)	162 (3)
O4′—H4′···O2^i^	0.84 (3)	1.85 (3)	2.673 (2)	165 (3)
O5—H5···O1	0.83 (3)	1.80 (3)	2.616 (3)	168 (3)
O5′—H5′···O2′	0.85 (4)	1.96 (4)	2.797 (3)	171 (4)
C10—H10*A*···O5′^ii^	0.97	2.60	3.380 (3)	138
C14—H14*B*···O1′^ii^	0.97	2.43	3.347 (3)	158
C14′—H14*D*···O2^iii^	0.97	2.36	3.241 (3)	151
C17′—H17′···O3′	0.93	2.49	3.326 (3)	149

Symmetry codes: (i) *x*−1/2, −*y*+1/2, *z*−1/2; (ii) −*x*+1, −*y*+1, −*z*+1; (iii) −*x*+1/2, *y*−1/2, −*z*+3/2.
